# Gut-Based Strategies to Reduce Postprandial Glycaemia in Type 2 Diabetes

**DOI:** 10.3389/fendo.2021.661877

**Published:** 2021-04-09

**Authors:** Md Kamruzzaman, Michael Horowitz, Karen L. Jones, Chinmay S. Marathe

**Affiliations:** ^1^ Department of Applied Nutrition and Food Technology, Islamic University, Kushtia, Bangladesh; ^2^ Adelaide Medical School, University of Adelaide, Adelaide, SA, Australia; ^3^ Endocrine and Metabolic Unit, Royal Adelaide Hospital, Adelaide, Australia

**Keywords:** gastric emptying, type 2 diabetes, postprandial glucose (PPG), incretin hormones, GLP-1 receptor agonist

## Abstract

Postprandial glycemic control is an important target for optimal type 2 diabetes management, but is often difficult to achieve. The gastrointestinal tract plays a major role in modulating postprandial glycaemia in both health and diabetes. The various strategies that have been proposed to modulate gastrointestinal function, particularly by slowing gastric emptying and/or stimulating incretin hormone GLP-1, are summarized in this review.

## Introduction

The importance of glycemic control to the optimal management of diabetes has now been clearly established (1). Glycemic control can be estimated in a number of ways – including random blood glucose, fasting glucose, postprandial glucose, oral glucose tolerance test or OGTT [which incorporates both fasting glucose and the glycemic response to an oral glucose load (usually 75g)] and glycated hemoglobin or HbA1c (which reflects overall glycaemia over a period of 8-12 weeks). Traditionally, the OGTT has been regarded as the ‘gold standard’ test for the diagnosis of diabetes, although HbA1c is increasingly used. Fasting glucose is used extensively for both diagnosis and monitoring of type 2 diabetes. Postprandial glycaemia, in contrast, has received relatively little attention, despite the recognition of its critical importance to overall glycaemia in type 2 diabetes, and probable relevance as an independent risk factor for macrovascular disease (2). Postprandial hyperglycemia is usually the first defect in glucose intolerance (3). Impaired glucose tolerance, defined as abnormal PPG (between 7.8-11.1mmol) in the presence of a normal fasting glucose i.e., specifically a postprandial glycemic abnormality, is regarded as a ‘pre-diabetic state’ predisposing to type 2 diabetes. In type 2 diabetes, PPG makes a substantial contribution to overall glycaemia, as measured by HbA1c, and is the dominant contributor (i.e. >50%) when the latter is ≤8.0% (4, 5). The significance of targeting PPG to achieve desirable glycemic goals has been increasingly appreciated in the last two decades. In 2001, the ADA published a consensus statement relating to PPG and subsequently, in 2014, the International Diabetes Federation (IDF) released specific strategies for the management of PPG excursions in type 1 and type 2 diabetes advocating the use of dietary strategies (such as low glycemic index foods) and use of anti-diabetic medications (such as GLP-1 agonists) which target postprandial glycaemia (2, 6). Postprandial hyperglycemia is not only associated with microvascular disease, but probably increases the risk of cardiovascular complications. The DECODE study reported that PPG predicted all-cause and cardiovascular mortality better than fasting plasma glucose (FPG) in type 2 diabetes (7).

## Gastrointestinal Determinants of Postprandial Glycemia

A number of factors impact postprandial glycemia. While these include pre-prandial glycemia, insulin secretion and sensitivity (hepatic and skeletal) and glucagon secretion this review focuses on gastrointestinal factors, particularly gastric emptying, intestinal carbohydrate absorption, and the incretin hormones gastric inhibitory polypeptide or glucose-dependent insulinotropic polypeptide (GIP) and glucagon-like peptide 1 (GLP-1) (6). The significance of the gastrointestinal tract in modulating postprandial glycemia is dependent on glucose tolerance status. One way of evaluating this contribution is by calculating so-called ‘gastrointestinal glucose disposal’ GIGD; the amount of intravenously administrated glucose required to ‘copy’ the glucose excursions after the oral glucose load - If 25g intravenous glucose is required to copy a 75g oral glucose load, GIGD amounts to 100 × (75 – 25)/75 = 66% (8, 9). In other words, in this instance, the gastrointestinal tract is able to dispose of 50g of glucose. In health, GIGD approximate 66%. However, in type 2 diabetes, GIGD is markedly reduced and may even be zero (10). Recent studies have provided important insights into the relevance of gastric emptying and the incretin hormones to GIGD.

## Gastric Emptying

Gastric emptying is the physiological process by which nutrients are transferred from the stomach to the duodenum at a tightly regulated rate to optimize their digestion and absorption (11). Gastric emptying is a complex coordinated process, involving the smooth muscle of the stomach, neural networks (Auerbach’s and Meissner’s plexi), vagal and enteric nervous systems, neurotransmitters such as nitric oxide, immune cells and the gastric ‘pacemaker cells’ known as the Interstitial cells of Cajal (ICC) (11). Ingested solid food is initially retained in the stomach while it is ground into small fragments, the majority <1mm in size, a process known as trituration. The food particles are then propelled across the pylorus into the duodenum, predominantly in a pulsatile manner. The overall rate of gastric emptying is dependent on both the composition and macronutrient content of a meal (12). Liquids are emptied preferentially when compared with solids. For solid emptying, there is typically an initial lag phase of about 20 min, while liquids empty essentially immediately. After the sloid lag phase gastric emptying of nutrient-containing foods (solid or liquid) typically approximates on overall linear pattern over time, whereas emptying of non-nutrient liquids follow a non-linear, volume-dependent, mono-exponential pattern. Accordingly, for nutrients, the volume of food ingested does not have a major impact the rate, as opposed to the duration, of emptying. In health, gastric emptying exhibits a wide inter-individual (about 1-3 kcal/min), but lesser intra-individual, variation (13). Abnormally delayed gastric emptying, or gastroparesis, occurs commonly in diabetes. Cross-sectional studies indicate that 30-50% patients with longstanding, complicated type 1 or 2 diabetes have gastroparesis. A hallmark of gastroparesis at the cellular level is loss of ICC (14). Conversely, gastric emptying may be accelerated in some people with diabetes, particularly well controlled uncomplicated type 2 diabetes (15) and adolescents with type 1 diabetes (16). Thus, the inter-individual variation in emptying is even wider in diabetes than health. Importantly in a given individual the rate of emptying, whether normal, delayed or more rapid, cannot be predicted based on clinical criteria. Whole upper gastrointestinal symptoms including postprandial fullness, nausea, vomiting, bloating, upper abdominal pain, and early satiety (17) are common in diabetes (18) and patients with gastroparesis often present with upper gastrointestinal symptoms, the relationship between the presence of symptoms and gastroparesis is modest at best.

Gastric emptying is a major determinant of postprandial glycemic excursions in both health and diabetes, accounting for a third of the variance in the initial rise in glucose. The relationship of the rate of gastric emptying to PPG is both time and glucose tolerance status-dependent (19). Accordingly, in health, following an oral glucose load.

The early (30 or 60 min) rise in plasma glucose is related directly to the rate of emptying, while the relationship to the 120 min value, which is used diagnostically is inverse, however in individuals with impaired glucose tolerance or type 2 diabetes, the relationship shows a ‘right-ward’ shift such that a direct relationship is observed even beyond 60 min (19, 20).

## Measurement of Gastric Emptying

Scintigraphy is the ‘gold standard’ technique of measuring gastric emptying and allows the precise measurement of both solid and liquid emptying, potentially simultaneously. The American Neurogastroenterology and Motility Society and the Society of Nuclear Medicine have proposed a test meal which contains the equivalent of two large eggs, two slices of bread and strawberry jam (30 g) with water (120 ml) and comprises 255 kcal (with a composition of 72% carbohydrate, 24% protein, 2% fat and 2% fiber). The meal is radiolabeled with 1mCi 99Tc sulfur colloid (21). This meal may be suitable for a Western diet, but its applicability globally is questionable. The limitations of scintigraphy relate to radiation exposure and the requirement for specialized nuclear medicine equipment and trained personnel. The best alternative is a stable isotope breath test which, while a notional rather than precise measurement, correlates reasonably with scintigraphy and is non-invasive technique without radiation exposure. Subjects consume a meal containing a ^13^C labelled substrate, which is enzymatically converted to ^13^CO_2_ in the liver and excreted through the lungs. Breath samples are collected for 2-4 hours postprandially. Ultrasound can also be used to measure emptying, but is observer dependent and requires highly trained personnel. One of the most common methods of measuring gastric emptying in clinical trials is using the plasma kinetics of oral paracetamol absorption. While inexpensive and simple, it is an imprecise technique that cannot be used to assess gastric emptying of solids and is not recommended (22). Single-photon emission computed tomography (SPECT), magnetic resonance imaging (MRI) and 3D ultrasound are also non-invasive procedures able to provide true 3D images of the effect of meals on gastric volume and gastric accommodation, but remain research techniques.

## Incretin Hormones

It has been known since the 1960’s that blood glucose levels are much lower following oral, compared with administration of a similar amount of intravenous glucose (23). This reflects the marked increase in insulin secretion following oral glucose, a phenomenon termed the ‘incretin effect’ (24). In late 1980s, the factors responsible for the incretin effect, the so-called ‘incretin’ hormones, glucose-dependent insulinotropic polypeptide (GIP) and glucagon-like peptide-1 (GLP-1) were discovered (25). GIP and GLP-1 are gut-derived peptides secreted from specialized entero-endocrine K (located predominantly proximally in the small intestine) and L (located predominantly more distally in the intestine) cells, respectively. All macronutrients have the capacity to stimulate incretin hormone secretion although their relative potency differs (fat and protein may be more powerful triggers for incretin release than carbohydrates). While low in the fasted state, plasma GLP-1 and GIP levels rise promptly following a meal (26). Circulating GLP-1 and GIP are rapidly degraded by the ubiquitous enzyme, dipeptidyl peptidase-IV (DPP-IV) and renal clearance, such that their half-lives are only a few minutes (27). The incretins both have glucose- dependent insulinotropic properties in health and GIP may be the dominant contributor to the incretin effect in health (28). GLP- 1 also slows gastric emptying substantially (whereas GIP has no effect) and suppresses glucagon, the latter in a glucose dependent manner (while GIP may stimulate it) (29, 30). A seminal observation was the recognition that the incretin effect is markedly reduced in type 2 diabetes (31). Incretin hormone secretion is essentially normal but the insulinotropic effect of GIP in type 2 diabetes is attenuated markedly (32). While that of GLP-1 is relatively maintained in type 2 diabetes. Intravenous infusion of GLP-1 in pharmacological concentrations reduces not only fasting, but also normalize postprandial glycemia in type 2 diabetes (33). Slowing of gastric emptying is a major mechanism to account for postprandial glucose lowering by exogenous GLP-1, as postprandial plasma insulin levels are usually reduced, rather than greater (34). These observations stimulated the development and subsequent widespread use of ‘GLP-1 based’ drugs for use in the management of type 2 diabetes. The latter are of two types: 1) GLP-1 receptor agonists and 2) DPP-IV inhibitors. Exenatide, the first GLP-1 receptor agonist, was approved by the FDA in 2005 and is a synthetic version of exendin-4, a peptide derived from saliva of the lizard, Heloderma suspectum, found to have ~50% similarity with human GLP-1, with resistance to DPP-IV degradation and relatively slow systemic clearance (35). Since then, a number of GLP-1 receptor agonists have been developed. These are currently administered subcutaneously once or twice a day (e.g., lixisenatide, liraglutide and exenatide BID) or once a week (e.g. exenatide QW, dulaglutide and semaglutide) (19, 35, 36). An oral formation of semaglutide has recently been developed (37, 38). Specific inhibitors of the DPP-IV enzyme prolong the availability of endogenous GLP-1 (and GIP) and are administered orally. A number of compounds in this class are available (e.g. sitagliptin, linagliptin, saxagliptin, vildagliptin and alogliptin) (19, 39, 40).

## Gut Based Management of Postprandial Glycemia (PPG)

**Figure 1:**
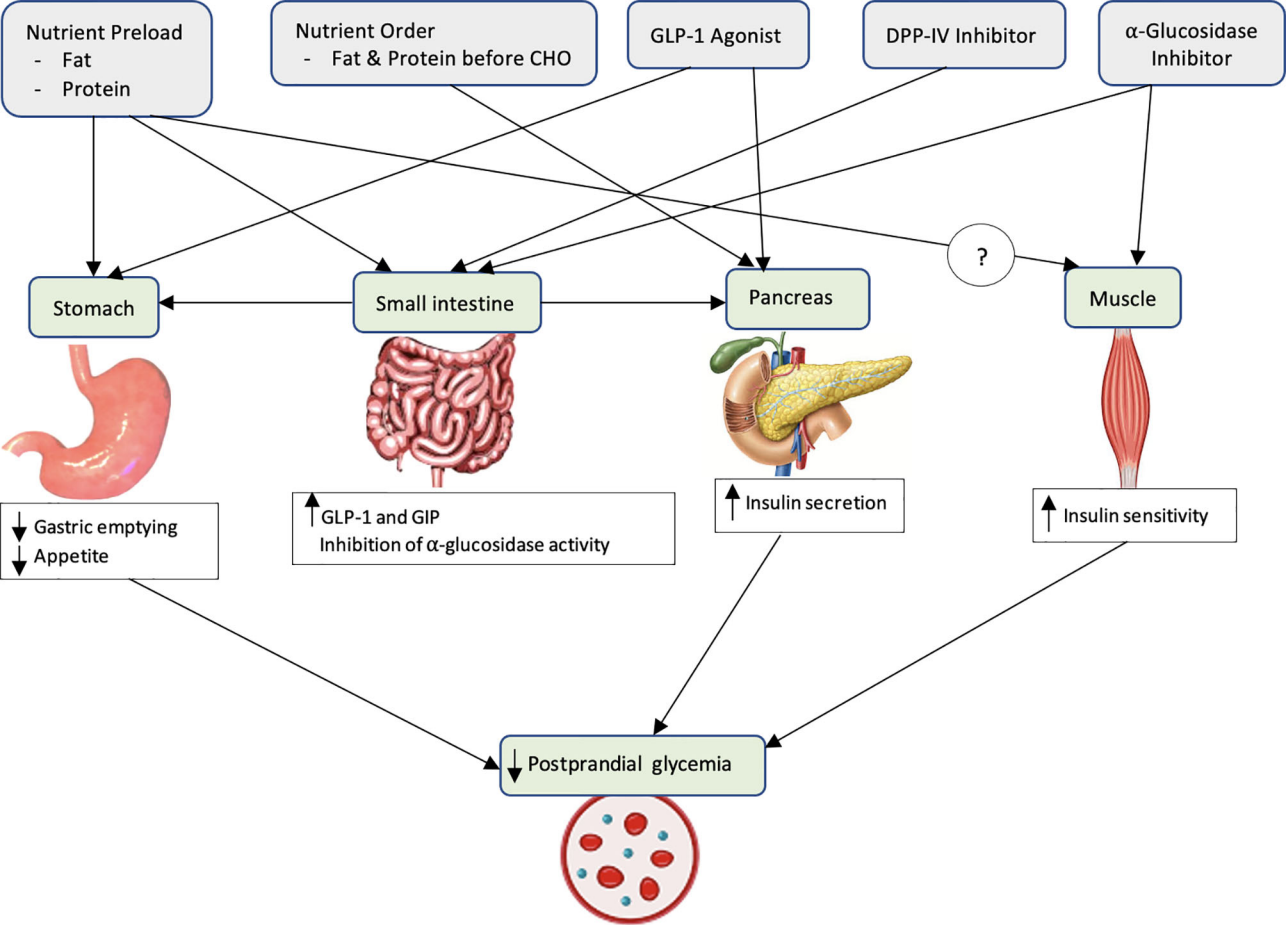
Potential mechanisms of postprandial glucose lowering of various gut-based strategies.

Several gut-based interventions/treatment strategies have been proposed to minimize postprandial glycemic excursions. These interventions can be broadly classified as i) dietary/non-pharmacological and ii) pharmacological (**Figure 1**).

### Dietary Approach

Nutritional/dietary management (**Table 1**) of postprandial hyperglycemia is an attractive option and underutilized. It may be of particular relevance to individuals with impaired glucose tolerance where pharmacological therapy is not usually mandatory.

**Table 1:** Dietary approaches to reduce postprandial glycaemia.

Strategy		Mechanism of action	Comments
***A. Nutrient Preload***	***Fat Preload*** Olive oil (41)	Delays gastric emptying. Triggers GLP-1 and GIP (~15 min post intervention) secretion.	Delayed postprandial glucose peak in type 2 diabetes. Involves extra energy intake. May cause gastrointestinal intolerance like nausea and vomiting. May be culturally unacceptable by individuals.
	***Protein*** Soy Protein (42) Whey Protein (42–45)	Delays gastric emptyingTriggers GLP-1, GIP and insulin secretion.	Reduced post meal glycaemia by 40-50% in type 2 diabetes. Less/No extra energy intake.
	***Artificial Sweetener*** Sucralose (46–49)	No impact on gastric emptying. No impact on postprandial glycaemia, GLP-1 or GIP secretion.	Studies performed in healthy individuals.Not tested in type 2 diabetes.
***B. Altering Macronutrient Composition***	***Protein*** (50–52)	Delays gastric emptying Triggers insulin secretion.	Reduced postprandial glycaemic excursion by 38-40%. Reduced HbA1c by 0.8-2.2% in type 2 diabetes.
	***Fat*** (53–56)	Delays gastric emptying. Increases insulin secretion and possibly, insulin sensitivity.	Delayed peak blood glucose in healthy subjects. Reduced postprandial glucose excursion. High fat may entail extra energy intake. May cause gastrointestinal intolerance e.g., nausea and vomiting.
	***Dietary* fiber** (57–63)	Delays gastric emptying. Increases early phase insulin secretion. Delays intestinal glucose absorption.	Reduced postprandial glycaemia by 35-43% in healthy subjects and type 2 diabetes. Reduced HbA1c level by ~0.5%. Dietary fiber does not involve additional energy intake.
***C. Altering Sequence of Macronutrient Consumption***	***Protein followed by Carbohydrates*** (64–69)	Delays gastric emptying. Increases release GLP-1, GIP, Cholecystokinin (CCK) and peptide YY. Delays carbohydrate absorption.	Reduced postprandial glycaemia by 39-50% in healthy subjects and type 2 diabetes. Reduced postprandial insulin excursion by ~25% in type 2 diabetes. Does not involve additional energy intake.
	***Protein and Fat followed by Carbohydrates*** (64, 70)	Delays gastric emptying rate. Triggers GLP-1 secretion.	Reduced postprandial glycaemic excursions by 78% and 60% after lunch and dinner respectively in type 2 diabetes. Reduced HbA1C by 0.3% in type 2 diabetes.

#### i) Nutrient Preload

**Figure 2:**
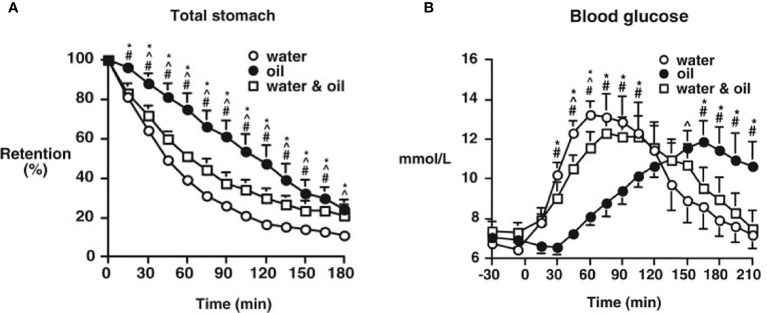
Gastric emptying **(A)** and blood glucose concentrations **(B)**, after ingestion of a mashed potato meal when either 30 ml olive oil (oil), 30 ml water (water), or 30 ml water with 30 ml olive oil (water and oil) was consumed 30 minutes before the meal by type 2 patients. Data are the mean ± SEM. *P < 0.05, oil vs. water; ^#^P < 0.05, oil vs. water and oil; ^P < 0.05, water vs. water and oil. [Reprinted with permission from Gentilcore et al. (41)].

A nutrient preload refers to consumption of a small amount of macronutrient at a fixed interval (30-60 min) before a meal to reduce the postprandial glycemic excursion. These nutrients may reduce postprandial glucose by a number of mechanisms including slowing gastric emptying, stimulating the release of incretins, and other gut peptides and reducing subsequent meal intake. Fat and protein have been best characterized as macronutrient preloads. Because of its higher-calorie content, fat is emptied from the stomach and absorbed relatively more slowly (71, 72) and GLP-1 and GIP secretion are triggered by a fat preload (73, 74). Ingestion of fat as a preload or direct small intestinal administration both slow gastric emptying and stimulate the release of GLP-1, effects mediated by the interaction of lipolytic products with the small intestine, and partly by CCK. The dominant effect of fat is likely to be *via* slowing of gastric emptying-this is analogous to the use of olive oil or equivalent to slow the absorption of alcohol-containing beverages (75) (**Figure 2**). Slowing of gastric emptying is not as marked when fat is mixed with other nutrients; due to low density, in the seated position fat may ‘layer’ on top of other nutrients and exert little impact initially on emptying (41, 73, 74). In both health and type 2 diabetes (42, 76) whey protein, a by-product of milk, has been the best characterized (43). Soy, or whey-based protein preloads may have a greater impact on postprandial glycemia than fat (42, 77). When a whey-based protein preload is taken 30 minutes before a large meal, there is a reduction in postprandial glucose of 28-50% is associated with increased secretion of insulin, GLP-1, and GIP (43, 44, 78). The effects of protein are hypothesized to be mediated primarily by amino acids (in the case of whey leucine, isoleucine, and valine). Whey protein is digested quickly, compared to other proteins such as casein, and is associated with substantial incremental rise in postprandial amino acids which triggers the release of both insulin and glucagon (79).

A potential limitation in the use of protein and fat preloads is that they provide additional energy intake in a group where obesity is very common. On the other hand, the preload has the potential to suppress subsequent energy intake. Consuming oil by itself before a meal, may also be associated with gastrointestinal intolerance, and is unlikely to be culturally acceptable widely. The characteristics of an ideal preload are that it should have the capacity to slow gastric emptying, reduce postprandial glycemia substantially and contain minimal calories. The preload should also be inexpensive, readily available, well tolerated and acceptable by the majority of individuals. The effects of low-calorie preloads have also been evaluated. The artificial sweetener sucralose (with no calories) has no impact on gastric emptying with little, if any, stimulation of GLP-1 [ (46–48). 3-O-methylglucose preload, a non-metabolized substrate of SGLT1, was reported to slow gastric emptying and stimulate GLP-1 and GIP, to reduce PPG excursions in the first 30 minutes after a meal, when compared with a glucose preload (46). The above studies, relating to the effects of nutrient preloads on postprandial glucose excursions, are limited to acute or short-term interventions, for a maximum period of five weeks. A longer-term intervention study reported a significant lowering of postprandial blood glucose (~14%) and HbA1c (0.3%), where participants with type 2 diabetes consumed Inzone Preload (consisting only of natural food ingredients including pea-protein, whey protein, egg albumin, Ω 3/6 fatty acids, whole eggs, apple, rosehip, and sugar beet fiber) (29% protein) 30 min before each of three meals daily for 12 weeks (80). Further studies are required.

#### ii) The Impact of Macronutrient Composition and Sequence on PPG

Macronutrient composition, sequence and timing of a meal can impact postprandial hyperglycemia by a number of mechanisms, which include slowing of gastric emptying. For example, fat and protein, are both inhibitors of gastric emptying. Accordingly, incorporation of protein and/or fat into a carbohydrate rich meal or changing the proportion of macronutrients has the potential to reduce PPG and thereby, HbA1c. Gannon et al. reported that increasing the ratio of protein and fat, while decreasing ratio of carbohydrate, leads to a 38% reduction in net mean 24-h integrated glucose area response (including PPG) (50, 81). Another study conducted by this group demonstrated that an increase in dietary protein from 15 to 30% is associated with a reduction in PPG of 40% and HbA1c of 0.8% in type 2 diabetes (51). The positive impact of protein incorporation is hypothesized to mainly reflect its direct stimulatory effect on insulin secretion and slowing of gastric emptying (82). The addition of fat has been shown to slow gastric emptying and reduce PPG levels (83), but, of necessity, increases the energy load substantially, which has the potential to affect glycemic regulation adversely (53, 84). For example, one study, in healthy and obese individuals, reported that an increase in fat intake increase plasma insulin, while reducing insulin sensitivity (54). In another study, in healthy subjects, consumption of high monounsaturated fat was associated with an improvement in insulin sensitivity when compared with a high-saturated-fat diet (53). Nevertheless, the positive effect of monounsaturated fat on insulin sensitivity were inconsistent when the proportion of energy derived from total fat surpassed 38% of total energy (53, 55, 85). Several studies conducted in healthy and type 2 diabetic individuals have shown that adding low glycemic index carbohydrates to the diet reduces PPG by ~40% and HbA1c levels by ~0.5% (57–60, 86–88), though the impact of a low GI or high-fiber diet is inconclusive (53). Therefore, replacing refined carbohydrates and added sugar with grains, legumes, vegetables, and fruits that are rich in dietary fiber may represent an efficient strategy to reduce PPG.

Another potential approach is to alter the sequence, or order of consumption of macronutrients during a meal. For example, initial ingestion of non-carbohydrate macronutrients and ingestion of carbohydrates last has been reported to be effective in reducing postprandial glucose excursions among individuals with type 2 diabetes and impaired or normal glucose tolerance (64, 89). For example, Shukla et al. reported that ingestion of protein and vegetables before carbohydrates leads to a 39% reduction in postprandial glycemia in individuals with impaired glucose tolerance (64). Likewise, consumption of meat or fish, or vegetables before carbohydrate has been reported to reduce the postprandial glucose peak by almost 50%, as well as delay it by 30–60min and raise the level of GLP-1 and GIP (65, 66). However, as well as slowing gastric emptying, Shukla et al. demonstrated that protein and fat initially leads to higher GLP-1 levels and slower carbohydrate absorption, and also suggested that fiber from vegetables may be responsible (64, 67). A number of other studies support the beneficial effect of fiber to lower postprandial glycemia (59, 61). As compared with the preload, this approach has the indirect advantage of not involving additional energy intake (70, 89). Along with a reduction of PPG excursions, intake of vegetables before carbohydrates may *per se* also reduce the risk of other metabolic disorders, including cardiovascular disease (90).

### Pharmacological Approach

#### GLP-1 Receptor Agonists

Based on their half-life, GLP-1 receptor agonists (**Table 2**) can be classified, as either ‘short-acting’, or ‘long-acting’. The ‘short-acting’ GLP-1 agonists, exenatide BID and lixisenatide, delay gastric emptying profoundly in a dose-dependent manner. The magnitude of the slowing of gastric emptying is also dependent on the baseline rate of emptying. It is not surprising, therefore, that ‘short acting’ GLP-1 agonists target postprandial hyperglycemia. It has been conventionally believed that long-acting GLP-1 receptor agonists, such as exenatide QW and liraglutide, are much more effective in targeting fasting, rather than postprandial, hyperglycemia and have little, if any, effect on gastric emptying. This concept was supported by the observation that continuous intravenous stimulation of the GLP-1 receptor is associated with tachyphylaxis i.e. lesser slowing of gastric emptying (91, 92, 115). However, it is now recognized that this concept is incorrect; both exenatide QW (116) and liraglutide (117) have been shown to slow emptying with sustained use, although the magnitude of their effect may be less than that of ‘short acting’ agonists. Given that modest slowing of emptying may have a major effect on postprandial glucose this is of relevance to their use in the management of type 2 diabetes (116).

**Table 2:** Summary of the pharmacological agents targeting postprandial glycaemia.

Class	Agent Name	Duration of action	Mode of administration	Mechanism of action	Comments
***Long acting GLP 1 Agonists*** (19, 22, 91–97)	Albiglutide Dulaglutide Exenatide XR Liraglutide Semaglutide	Half-life few days	Subcutaneous injection (Once daily; once weekly)	Slows gastric emptying and increase satiety.Increases glucose-dependent insulin secretion. Reduces glucose-dependent glucagon secretion.	Less impact on gastric emptying compared to short-acting GLP-1 Agonists. More effective in controlling fasting/preprandial hyperglycaemia.
***Short acting GLP-1 Agonists*** (19, 22, 91, 92, 94, 95)	Exenatide BIDLixisenatide	Half-life 2.4 to 8 hours	Subcutaneous injection (Once or twice daily)	Delays gastric emptying and increase satiety. Increases glucose-dependent insulin secretion. Reduce glucose-dependent glucagon secretion.	More effective in slowing gastric emptying and controlling postprandial hyperglycaemia compared to long-acting GLP-1 Agonists. Gastrointestinal intolerance e.g., nausea, vomiting, diarrhea, may limit uses.
***GIP receptor Agonists and*** ***Antagonists*** (98, 99)	AC163794 (Pro^3^)GIP	Long acting(>24 hours)	Subcutaneous injection (Once daily)	Enhances insulin secretion.	Experiments performed only in mice models and show significant reduction in overall hyperglycaemia. Data evaluating postprandial response not available. Human trials are awaited.
***GIP/GLP-1 receptor Agonists*** (22, 100–103)	Tirzepatide (LY3298176)	Long Acting(5 Days)	Subcutaneous injection (Once weekly)	Combined action of both GLP-1 and GIP as above.	Impressive dose-dependent reduction in overall glycaemia in type 2 diabetes. Data evaluating postprandial response not available.
***DPP-4 inhibitors*** (19, 39, 40, 94, 95, 104–106)	Alogliptin Linagliptin Saxagliptin SitagliptinVildagliptin	Long acting (half-life 3 to > 22 hours)	Oral (Once or twice daily)	Prevent degradation of GLP-1 and GIP *in vivo*, prolonging its action.	Has little or no effect on gastric emptying and satiety. Modest effect to reduce postprandial hyperglycaemia.
***Amylin Agonist***(19, 95, 107–109)	Pramlintide	Short-acting(half-life ~48 min)	Subcutaneous injection (Three times daily)	Delays gastric emptying and increases satiety.Reduces glucagon secretion.	Modest effect to reduce postprandial hyperglycaemia. Gastrointestinal intolerance e.g., nausea, vomiting. Required to adjust insulin dose to avoid hypoglycemia.
**Alpha-glucosidase inhibitors** (95, 110–114)	Acarbose MiglitolVoglibose	Short-acting(half-life ~2 to 4 hours)	Oral administration(Three times daily)	Inhibit maltase-glucoamylase intestinal (α-glucosidase) and pancreatic α-amylase which in turns delay gastric emptying, carbohydrate absorption and prompt GLP-1 release.	Reduce postprandial glucose and HbA1C. Gastrointestinal adverse effects common.

Some limitations of the strategy of using GLP-1 agonists for modulating gastric emptying and enhancing incretin secretion should be recognized: i) GLP-1 agonists should probably not be used in patients with existing gastroparesis. In the LEADER study, ‘delayed gastric emptying’ was reported three times more frequently in the liraglutide than the placebo group (93) ii) Gastrointestinal adverse effects, particularly nausea, vomiting and diarrhea, may limit tolerability. Unfortunately, in the majority of studies these have been assessed by participant self-report which is known to be unreliable (118) iii) the insulinotropic actions of GLP-1-based therapy necessitates adequate endogenous insulin secretory capacity iv) GLP-1 agonists are contraindicated in the rare case of medullary thyroid carcinoma. While recent observational studies and meta-analyses have failed to establish a causal relationship between GLP-1 agonists and acute pancreatitis, this remains potential issue (94, 119). A post-hoc analysis of the LEADER trial showed that the GLP-1 receptor agonist, liraglutide, to have an increased risk of gallbladder or biliary tract related events compared with placebo (120). It should be noted, however, that this trial was not specifically designed to assess the risks of gallbladder event rates with liraglutide.

#### GIP-Based Medications

There is some renewed interest in GIP-based drugs, especially when co-administered with GLP-1 (GIP/GLP-1 co-agonists) with studies suggesting that both GIP agonism and GIP antagonism may facilitate weight loss in type 2 diabetes. The effects of GIP-based drugs on gastrointestinal motility remain to be studied. However, given that GIP does not impact gastric emptying, it appears unlikely that an effect of GIP agonism is unlikely (22, 100).

#### DPP-IV Inhibitors

DPP-IV inhibitors (e.g. sitagliptin, vildagliptin, saxagliptin, linagliptin, and alogliptin) reduce both pre- and postprandial glucose (95) but have minimal, if any, effect on gastric emptying (104). Postprandial glucose-lowering by DPP-IV inhibitors may, however, be potentiated by a nutrient preload (121) a strategy which warrants additional exploration (105).

#### Acarbose

Acarbose, and other alpha-glucosidase inhibitors, such as voglibose, delays the production of monosaccharides from complex carbohydrates by inhibiting the alpha-glucosidase on the brush border membrane of the small intestine (122) to diminish postprandial glucose excursions (110, 122, 123). However gastrointestinal adverse effects are common (111).

#### Pramlintide

The synthetic analog of amylin, which is co-secreted with insulin in the pancreatic beta cells, pramlintide, reduces postprandial glucose in part by delaying gastric emptying. It may also enhance satiety (107, 108, 124).

## Conclusions

Modulating gastrointestinal motility (especially slowing gastric emptying) and stimulating the incretin system are major targets for management of postprandial glycemia. A number of strategies, dietary as well as pharmacological, have been introduced. Regarding simple dietary approaches have shown promising results in small studies and larger trials are required to establish efficacy. On the other hand, pharmacological strategies, such as GLP-1 agonists are prescribed widely for type 2 management, but their use is largely empirical. There is a need for studies to evaluate their efficacy under various glycemic conditions and the relationship of the effects of these drugs on glycemic control with those on gastric emptying in an attempt to provide more targeted and personalized management.

## Author Contributions

All authors contributed to the article and approved the submitted version.

## Conflict of Interest

The authors declare that the research was conducted in the absence of any commercial or financial relationships that could be construed as a potential conflict of interest.
